# Clinical Practices and Institutional Protocols on Prophylaxis, Monitoring, and Management of Selected Adverse Events Associated with Trastuzumab Deruxtecan

**DOI:** 10.1093/oncolo/oyac107

**Published:** 2022-06-01

**Authors:** Aditya Bardia, Kathleen Harnden, Lauren Mauro, Angela Pennisi, Melissa Armitage, Hatem Soliman

**Affiliations:** Massachusetts General Hospital Cancer Center, Harvard Medical School, Harvard University, Boston, MA, USA; Breast Oncology Program, Inova Schar Cancer Institute, Fairfax, VA, USA; Breast Oncology Program, Inova Schar Cancer Institute, Fairfax, VA, USA; Breast Oncology Program, Inova Schar Cancer Institute, Fairfax, VA, USA; Department of Breast Oncology, H. Lee Moffitt Cancer Center & Research Institute, Tampa, FL, USA; Department of Breast Oncology, H. Lee Moffitt Cancer Center & Research Institute, Tampa, FL, USA; Experimental Therapeutics Program, H. Lee Moffitt Cancer Center & Research Institute, Tampa, FL, USA

**Keywords:** breast neoplasm, clinical practice patterns, drug-related side effects and adverse reactions, trastuzumab deruxtecan

## Abstract

The treatment of metastatic breast cancer (mBC) has evolved significantly in the past several years with the approval of new targeted agents. Trastuzumab deruxtecan (T-DXd), an antibody-drug conjugate with a topoisomerase I inhibitor payload, is a new addition to the class of therapies that target the human epidermal growth factor 2 (HER2) receptor. T-DXd was approved in the US in December 2019 for patients with HER2-positive metastatic or unresectable breast cancer who have received 2 or more prior anti-HER2–based regimens in the metastatic setting. In the DESTINY-Breast01 phase II trial (NCT03248492), T-DXd demonstrated high rates of durable responses in heavily pretreated patients with HER2-positive mBC, with a confirmed objective response rate of 62%, median duration of response of 18.2 months, and median progression-free survival of 19.4 months. In addition to efficacy, successful implementation of any new anticancer therapy includes learning how to prevent, monitor, and manage treatment-related adverse events. As T-DXd becomes more widely used, information can be gained from real-world clinical practices, institutional approaches, and the collaboration of multidisciplinary oncology teams who treat patients with T-DXd. This article reviews practical insights and management of nausea and vomiting, neutropenia, interstitial lung disease, risk of cardiotoxicity, and other adverse events associated with T-DXd administration from the perspective of health care providers who have experience utilizing T-DXd.

## Teaser

This article reviews practical insights and management of nausea and vomiting, neutropenia, interstitial lung disease, risk of cardiotoxicity, and other adverse events associated with trastuzumab deruxtecan (T-DXd) administration from the perspective of health care providers .who have experience utilizing T-DXd

**Implications for Practice**

Trastuzumab deruxtecan (T-DXd) is one of the latest additions to the treatment armamentarium for human epidermal growth factor 2 (HER2)–positive metastatic breast cancer. T-DXd is a novel targeted therapy that utilizes antibody-drug conjugate technology to deliver a topoisomerase I inhibitor payload directly to cancer cells, causing DNA damage and cell apoptosis. T-DXd has demonstrated efficacy in hard-to-treat patients and has an adverse event (AE) profile that can generally be managed with awareness and education. Guidance on AEs is provided in the Warnings and Precautions section of the Prescribing Information, but additional information can be garnered from real-world clinical practices and institutional protocols from clinicians with experience administering T-DXd.

## Introduction

Approximately 15%-20% of patients with breast cancer (BC) have tumors that overexpress human epidermal growth factor 2 (HER2), or HER2-positive BC,^1-3^ which is associated with an aggressive clinical phenotype and a historically poor prognosis. In recent years, however, HER2-positive BC has shifted to being a disease that is highly treatable owing to the development and clinical integration of HER2-targeted therapies. Today, prolonged survival is possible even in patients with metastatic disease. In a study examining patients with stage I and II BC treated between 1975 and 1981, those with HER2-positive disease had the worst survival rates regardless of hormone receptor status.^4^ Decades later, a study that evaluated patients with metastatic BC (mBC) diagnosed between 2004 and 2007 found that patients with HER2-positive disease had the most favorable survival rates.^5^ In addition to managing the disease, an important part of the mBC treatment plan consists of preventing, monitoring for, and managing treatment-related adverse events (AEs). This becomes even more significant as a greater number of treatments are approved, and their use becomes widespread.

## Trastuzumab Deruxtecan

In recent years, new targeted therapy options for the treatment of patients with HER2-positive mBC have gained rapid approvals. These approved HER2-targeted therapies include monoclonal antibodies, tyrosine kinase inhibitors, and antibody-drug conjugates. Trastuzumab deruxtecan (T-DXd) is a HER2-directed antibody-drug conjugate that was approved in the US in December 2019 for the treatment of patients with HER2-positive metastatic or unresectable BC who have received 2 or more prior anti-HER2–based regimens in the metastatic setting. The HER2 antibody component of T-DXd consists of a human monoclonal immunoglobulin G1 produced with reference to the same amino acid sequence as trastuzumab.^6^ The molecule includes a topoisomerase I inhibitor payload, DXd, that is attached to the antibody by a tumor-selective cleavable linker. T-DXd binds to HER2 on tumor cells, internalizes, and is cleaved by lysosomal enzymes. Upon release, the membrane-permeable T-DXd causes DNA damage and apoptotic cell death. The payload is also membrane permeable, which enables a bystander effect, resulting in the elimination of both target and surrounding tumor cells.^6-8^

DESTINY-Breast01 (NCT03248492) is an open-label, international, multicenter, phase 2 study of T-DXd in patients with HER2-positive mBC^9^ that supported the regulatory approval of T-DXd in the US in 2019, followed by other global approvals.^10^ Updated longer-term efficacy results from the March 2021 data analysis were recently presented and are summarized in Table 1. Consistent with prior results, T-DXd provided patients with high rates of durable responses, with a confirmed objective response rate (ORR) of 62% (95% CI, 54.5%-69.0%), the median duration of response (DOR) of 18.2 months (95% CI, 15.0 months-NE), median progression-free survival (PFS) of 19.4 months (95% CI, 14.1-25.0 months), and median overall survival of 29.1 months (95% CI, 24.6-36.1 months) in a heavily pretreated population of patients with HER2-positive mBC (median duration of follow up 26.5 months).^11^ Most recently, the phase III DESTINY-Breast03 (NCT03529110) trial in patients with HER2-positive mBC demonstrated that treatment with T-DXd resulted in significantly longer median PFS of (not reached [95% CI, 18.5 months-NE]) compared to 6.8 months (95% CI, 5.6-8.2 months) (hazard ratio 0.28 [95% CI, 0.22-0.37]) than in patients treated with T-DM1 in the second or greater line setting (median duration of follow-up was 16.2 months for T-DXd and 15.3 months for T-DM1).^12^

**Table 1.** DESTINY-Breast01 March 2021 efficacy and safety results^11^

Efficacy data	
Patients	253 patients, who were required to have mBC that progressed on or after T-DM1, were enrolled; 184 patients received T-DXd 5.4 mg/kg, representing the primary analysis set
Duration of follow-up, months, median (range)	26.5 (0.7-39.1)
Patients who remained on treatment, *n* (%)	28 (15.2)
Confirmed ORR (primary endpoint)	62% (95% CI, 54.5%-69.0%)
Median DOR	18.2 months (95% CI, 15.0 months-NE)
Median PFS	19.4 months (95% CI, 14.1-25.0 months)
Median OS	29.1 months (95% CI, 24.6-36.1 months)
**Safety data**	
Patients with a TEAE, *n* (%)	183 (99.5)
Grade ≥3 TEAE, *n* (%)	116 (63.0)
TEAE associated with discontinuation, *n* (%)	35 (19.0)
TEAE associated with death, *n* (%)	10 (5.4)
Drug-related ILD per ILD adjudication committee, *n* (%)	29 (15.8)
Drug-related ILD per grade, per ILD adjudication committee,^a^*n* (%)	Grade 1: 7 (3.8)
	Grade 2: 16 (8.7)
	Grade 3: 1 (0.5)
	Grade 5: 5 (2.7)

At data analysis, 1 grade 1 event and 1 grade 3 events were pending adjudication.

Abbreviations: DOR, duration of response; ILD, interstitial lung disease; mBC, metastatic breast cancer; ORR, objective response rate; OS, overall survival; PFS, progression-free survival; T-DM1, ado-trastuzumab emtansine; T-DXd, trastuzumab deruxtecan; TEAE, treatment-emergent adverse event.

The most common AEs included gastrointestinal AEs, specifically nausea and vomiting (N/V), and hematologic toxicity.^9^ Additional AEs of interest are those associated with other currently available HER2-targeted agents. For example, the warnings and precautions associated with trastuzumab and ado-trastuzumab emtansine (T-DM1) include decreased left ventricular ejection fraction (LVEF) and pulmonary toxicity, and with trastuzumab, neutropenia.^13,14^ However, unlike with other HER2-targeted therapies,^13,15,16^ clinically significant cardiomyopathy associated with T-DXd, particularly left ventricular dysfunction, was infrequently observed, with a total of 4 events of LVEF decrease (3 grade 2 and 1 grade 3) observed in the June 2020 data analysis of DESTINY-Breast01.^17^ In addition, although pulmonary toxicity is a risk with other HER2-targeted agents,^13,14^ T-DXd has demonstrated notable rates of interstitial lung disease (ILD) in clinical studies, with several fatalities.^9^ In the latest DESTINY-Breast01 data analysis (March 2021), 15.8% of patients (*n* = 29) experienced ILD, with 2.7% (*n* = 5) classified as grade 5 drug-related ILD (per the ILD adjudication committee).^11^ Therefore, monitoring for and managing these AEs are essential components of T-DXd therapy.

Because T-DXd was recently approved and has a novel mechanism of action, a better understanding of AE monitoring and management is needed to ensure that patients fully benefit from this therapy. The Prescribing Information provides recommendations on managing certain AEs based on clinical trial evidence (Table 2); however, the real-world clinical experience can bridge the gap between clinical research and practice. Information can be gained from the clinical practices of different members of the multidisciplinary oncology teams that represent various institutions around the US. This paper represents the perspectives of 5 oncologists and 1 clinical pharmacist who represent 3 institutions and have clinical experience treating mBC patients with T-DXd.

**Table 2.** T-DXd dosage modifications for AEs recommended in the prescribing information

AE	Severity/reaction		Dosage or schedule modification for T-DXd and additional recommendations
Neutropenia^7^	Grade 3 (<1.0-0.5 × 10^9^/L)		Interrupt T-DXd until resolved to grade 2 or less, then maintain dosage
	Grade 4 (< 0.5 × 10^9^/L)		Interrupt T-DXd until resolved to grade 2 or less Reduce dosage by 1 level^a^
Febrile neutropenia^7^	Absolute neutrophil count of < 1.0 × 10^9^/L and temperature greater than 38.3 °C or a sustained temperature of 38 °C or greater for more than 1 hour		Interrupt T-DXd until resolved Reduce dosage by 1 level^a^
Thrombocytopenia^7^	Grade 3 (platelets < 50-25 × 10^9^/L)		Interrupt T-DXd until resolved to grade 1 or less, then maintain dosage
	Grade 4 (platelets < 25 × 10^9^/L)		Interrupt T-DXd until resolved to grade 1 or less Reduce dosage by 1 level^a^
ILD^9^	Grade 1		• he administration of T-DXd must be interrupted. T-DXd can be restarted only if the event is fully resolved: ◦ If resolved in ≤ 28 days from day of onset, maintain dosage ◦ If resolved in > 28 days from day of onset, reduce dosage by 1 level. However, if the event of grade 1 ILD occurs beyond cycle day 22 and has not resolved within 49 days from the last infusion, the drug should be discontinued • Monitor and closely follow up in 2-7 days for onset of clinical symptoms and pulse oximetry • Consider follow-up imaging in 1-2 weeks (or as clinically indicated) • Consider starting systemic corticosteroids (eg, at least 0.5 mg/kg/day of prednisone or equivalent) until improvement, followed by gradual taper over ≥4 weeks • If worsening of diagnostic observations despite initiation of corticosteroids, then follow grade 2 guidelines. (If the patient is asymptomatic, then they should still be considered as grade 1 even if corticosteroid treatment is given)
	Grade 2		• Permanently discontinue patient from T-DXd treatment • Promptly start systemic corticosteroids (eg, at least 1 mg/kg/day of prednisone or equivalent) for at least 14 days or until clinical improvement, followed by gradual taper over ≥4 weeks • Monitor symptoms closely • Reimage as clinically indicated • If worsening or no improvement in clinical or diagnostic observations in 5 days, consider increasing dosage of corticosteroids (eg, 2 mg/kg/day of prednisone orequivalent) and administration may be switched to intravenous (eg, methylprednisolone) • Reconsider additional workup for alternative etiologies as described above • Escalate care as clinically indicated
	Grade 3 and 4		• Permanently discontinue patient from T-DXd treatment • Hospitalization required • Promptly initiate empiric high-dose methylprednisolone IV treatment (eg, 500-1000 mg/day for 3 days), followed by ≥1.0 mg/kg/day of prednisone (or equivalent) for at least 14 days until clinical improvement, followed by gradual taper over ≥4 weeks • Reimage as clinically indicated • If still no improvement within 3-5 days: • Reconsider additional workup for alternative etiologies as described above • Consider other immunosuppressants and/or treat per local practice
Decreased LVEF^7^	LVEF > 45% and absolute decrease from baseline is 10% to 20%		Continue treatment with T-DXd
	LVEF 40% to 45%	And absolute decrease from baseline is < 10%	Continue treatment with T-DXd Repeat LVEF assessment within 3 weeks
		And absolute decrease from baseline is 10% to 20%	Interrupt T-DXd Repeat LVEF assessment within 3 weeks If LVEF has not recovered to within 10% from baseline, permanently discontinue T-DXd If LVEF recovers to within 10% from baseline, resume treatment with T-DXd at the same dosage
	LVEF < 40% or absolute decrease from baseline is > 20%		Interrupt T-DXd Repeat LVEF assessment within 3 weeks If LVEF of <40% or absolute decrease from baseline of >20% is confirmed, permanently discontinue T-DXd
	Symptomatic congestive heart failure		Permanently discontinue T-DXd

T-DXd dosage reduction schedule for breast cancer: recommended starting dosage, 5.4 mg/kg; first dosage reduction, 4.4 mg/kg; second dosage reduction, 3.2 mg/kg; requirement for further dosage reduction, discontinue treatment.

Abbreviations: AE, adverse event; ILD, interstitial lung disease; IV, intravenous; LVEF, left ventricular ejection fraction; T-DXd, trastuzumab deruxtecan.

In this review, we will discuss the practices and insights learned from treating patients with T-DXd and will specifically review the AE risk management of N/V, neutropenia, ILD, and LVEF, along with some additional AEs of interest.

## Patient Counseling on AEs Prior to T-DXd Treatment

As with other anticancer therapies, patient counseling on T-DXd treatment prior to administration is an important component in establishing treatment expectations and empowering patients to actively participate in the management of their disease. AE counseling typically includes a discussion on N/V and the risk for neutropenia, ILD, and cardiotoxicity. N/V is a side effect observed with T-DXd; however, it can be effectively managed with proper use of prophylactic antiemetics, both before and after T-DXd infusion. Patients should be advised to promptly report symptoms of N/V so that antiemetic therapy can be escalated if needed. As previously mentioned, ILD is also an AE that can occur with T-DXd, and fatal cases have been observed in clinical trials. Therefore, this warrants patient education on monitoring for signs and symptoms of ILD including cough, shortness of breath, fever, or any new and worsening respiratory symptoms.^7^ A discussion of ILD and the other identified AEs is presented in more detail in the sections that follow.

## Nausea and Vomiting

**Figure 1.**
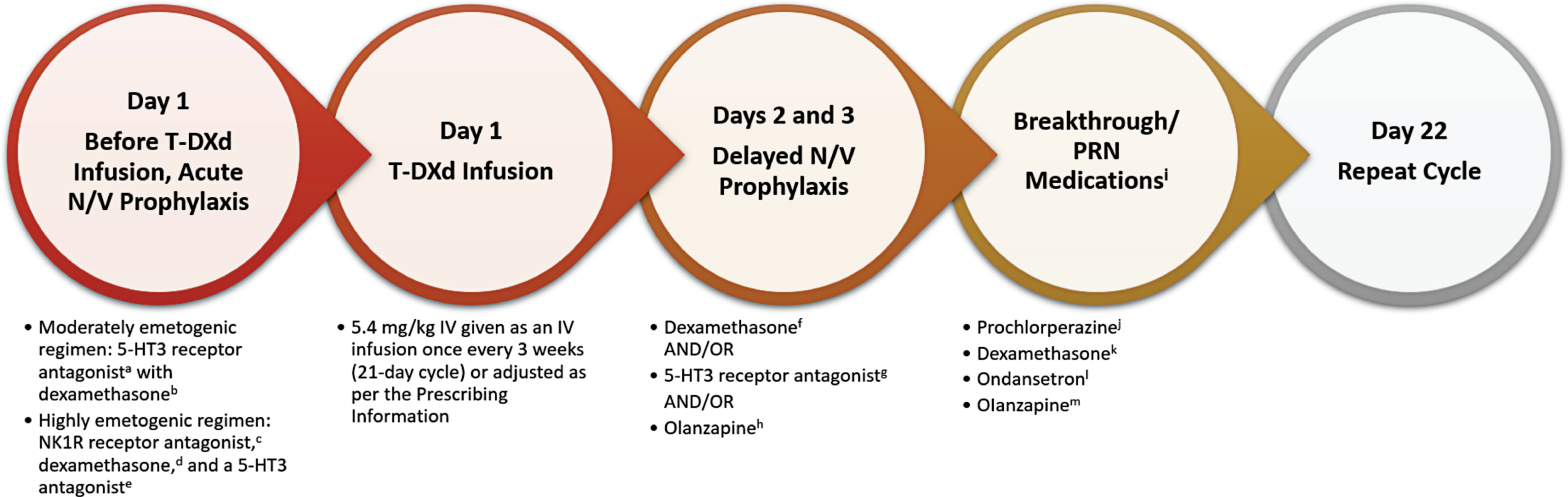
Prophylactic and management strategies for nausea and vomiting associated with T-DXd therapy. ^a^5-HT3 receptor antagonist (choose one).^18^ • Dolasetron 100 mg PO once. • Granisetron 10 mg SC once or 2 mg PO once or 0.01 mg/kg (max. 1 mg) IV once or 3.1 mg/24-hour transdermal patch applied 24 to 48 hours prior to first dose of T-DXd. • Ondansetron 16 mg to 24 mg PO once or 8 mg to 16 mg IV once. • Palonosetron 0.25 mg IV once. ^b^Dexamethasone 12 mg PO/IV once.^18^ ^c^May add NK1R antagonist (choose one). • Aprepitant 125 mg PO once. • Aprepitant injectable emulsion 130 mg IV^19^ • Fosaprepitant 150 mg IV once. • Netupitant 300 mg/palonosetron 0.5 mg (available as fixed-combination product only) PO once. • Fosnetupitant 235 mg/palonosetron 0.25 mg (available as fixed-combination product only) IV once. • Rolapitant 180 mg PO once on days 2 and 3. ^d^Dexamethasone 12 mg IV.^19^ ^e^Ondansetron 16 mg IV.^19^ ^f^Dexamethasone 8 mg PO/IV once on days 2 and 3.^18^ ^g^5-HT3 receptor antagonist.^18^ • Granisetron 1 mg to 2 mg (total dosage) PO daily or 0.01 mg/kg (max. 1 mg) IV SC once or 2 mg PO once or 0.01 mg/kg (max. 1 mg) IV once on days 2 and 3. • Ondansetron 8 mg PO once (twice daily) or 16 mg PO daily or 8 mg to 16 mg IV daily on days 2 and 3. • Dolasetron 100 mg PO daily on days 2 and 3. ^h^Olanzapine 5 mg to 10 mg PO daily on days 2 and 3.^18^ ^i^Lorazepam 0.5 mg to 2 mg PO beginning on the night before treatment and repeated the next day 2 hours before anticancer therapy begins to manage anticipatory N/V,^18^ or 0.5 mg to 1 mg PO Q6H PRN. Breakthrough N/V options: ^j^Prochlorperazine 10 mg IV/PO Q6H PRN for N/V. ^k^Dexamethasone 4 mg to 8 mg BID. ^l^Ondansetron 8 mg PO every 8 to 12 hours (16 mg-24 mg total daily dosage or 8 mg-16 mg IV). ^m^Olanzapine 2.5 mg to 10 mg PO daily. Abbreviations: BID, twice a day; IV, intravenous; NK1R, neurokinin-1 receptor; N/V, nausea/vomiting; PO, by mouth; PRN, as needed; Q6H, every 6 hours; SC, subcutaneous; T-DXd, trastuzumab deruxtecan.

Although the majority of patients in the August 2019 data analysis of the DESTINY-Breast01 trial reported N/V during treatment with T-DXd (77.7% nausea and 45.7% vomiting of any grade), the trial did not specify what antiemetic regimen was administered.^9^ In the clinical practice of the authors represented here, N/V associated with T-DXd can be effectively managed and often eliminated through antiemetic premedication use (Fig. 1).

The National Comprehensive Cancer Network guidelines classify T-DXd as a moderately emetogenic agent.^18^ The experience of 10 patients treated with T-DXd at Inova Schar Cancer Institute showed that a number of those patients had grade 1 or 2 N/V despite pretreatment with moderately emetogenic regimens (typically a combination of dexamethasone 8 mg or 12 mg and ondansetron 16 mg, with the addition of diphenhydramine 12.5 mg or 25 mg in 2 cases). Supportive care in oncology has evolved so much that patients with cancer should no longer endure N/V as an expected AE. As such, T-DXd was reclassified by this institution as a highly emetogenic agent. A neurokinin-1 receptor antagonist (aprepitant) has been added to the ondansetron and dexamethasone premedication regimen (Fig. 1), which has resulted in reduced N/V.^19^ The standard breakthrough/as needed medications (eg, prochlorperazine, dexamethasone, ondansetron, olanzapine; see Fig. 1) are still used at home for breakthrough symptoms but are needed less frequently given the adjusted antiemetic premedication regimen.

When selecting antiemetic regimens, these authors consider previous chemotherapy tolerability and other patient-specific factors. Patient-specific general risk factors for the development of N/V include young age, female sex, history of motion sickness, and/or pregnancy-related N/V, and history of emesis with prior anticancer agents. Considering these factors, antiemetic medications may be adjusted, and the regimen can be further tailored to the specific patient.^18^ For example, patients who are more prone to nausea based on their treatment history or who have a history of significant motion sickness may benefit from utilizing nightly olanzapine (typically on days 1-4 of each cycle) during T-DXd treatment. Furthermore, other patient-specific factors may require adjustment of the antiemetic including concomitant use of other QTc-prolonging medications or psychiatric medications, history of constipation, or migraines may be worsened by some of the aforementioned antiemetics.

A neurokinin-1 receptor antagonist (eg, fosaprepitant) is often added as a chemotherapy premedication if the patient experiences N/V after standard premedication with a 5-HT3 receptor antagonist (eg, ondansetron, palonosetron) and dexamethasone. Lorazepam can be added for patients who experience anticipatory N/V, but this is rarely needed in the practices represented here. Utilization of T-DXd dosage reductions is not typically needed to control N/V, but if N/V becomes severe, the dosage adjustment recommendations in the Prescribing Information (Table 2, footnote a) can be followed. With proactive measures and proper vigilance, N/V can be effectively managed.

## Neutropenia

Of the patients administered T-DXd in the DESTINY-Breast01 August 2019 data analysis, 21.2% (*n* = 39) demonstrated neutropenia of any grade, (6.5% [*n* = 12] grades 3-4), as reported by investigators. Three patients (1.6%) had febrile neutropenia.^9^ Clinical trials and real-world experiences at our institutions indicate that neutropenia is commonly encountered with T-DXd administration. It is important to counsel patients on the risk of this AE prior to initiating T-DXd treatment. Patients should be informed that neutropenia will be monitored via blood draws prior to each T-DXd dose and as clinically indicated.^9^ They should be advised that the T-DXd dose could be held or reduced, and/or supportive treatment with a granulocyte colony-stimulating factor could be added if neutropenia develops. Patients should also be aware that infection can occur, and they should promptly notify the treatment team in the event of a fever.

Neutropenia can be managed through dosage interruptions and reductions, as detailed in the Prescribing Information (Table 2). Specific recommendations on the use of growth factor support are not available and are usually determined based on patient-specific factors. Heavily pretreated patients may have depleted bone marrow reserve, which can impact the efficacy of growth factors; in this scenario, T-DXd dosage reductions may be a more effective approach or may be required in addition to growth factor use. If the patient is due for the next T-DXd cycle but absolute neutrophil count (ANC) remains significantly less than the treatment threshold of 1000/mm^3^ (grade 3/4 neutropenia), then treatment should be delayed a few days until it has resolved to grade 2 neutropenia. Repeat complete blood counts are performed to confirm ANC recovery prior to resuming therapy. In the event of grade 4 neutropenia, the T-DXd dose should be reduced by one dose level upon restart.^7^

Patients who need a rapid antitumor response may benefit more from the addition of growth factors support than from dosage alterations so as not to decrease the effectiveness of the therapy. In the clinical experience of the practitioners represented here, the addition of a short-acting growth factor (eg, filgrastim 5 μg/kg subcutaneously daily^20^) for 2-3 days can be an effective and quick way to elevate white blood cell (WBC) count and allow the patient to subsequently receive T-DXd once ANC increases to more than 1000/mm^3^. In addition, a long-acting growth factor (eg, pegfilgrastim 6 mg subcutaneously) may also be added on day 2 to avoid delays like this in future cycles.^21^ If ANC is near 1000/mm^3^ at the time of the next cycle, then treatment may be able to be continued on schedule with the addition of a long-acting growth factor, as described above. When long-acting growth factors are added, they are typically continued for the remainder of the treatment course.

Pharmacists are an important resource and can assist in selecting the appropriate WBC growth factor for the patient, considering patient and insurance company preferences. Some patients may have experienced unpleasant AEs (eg, headaches, muscle/joint aches) with growth factor use and may prefer a dosage reduction of T-DXd. Other patients may tolerate short-acting growth factors better than long-acting growth factors. When neutropenia develops at this advanced stage of mBC, the benefits of aggressive management should be weighed against the tolerability concerns for growth factor support.

## Interstitial Lung Disease

**Figure 2.**
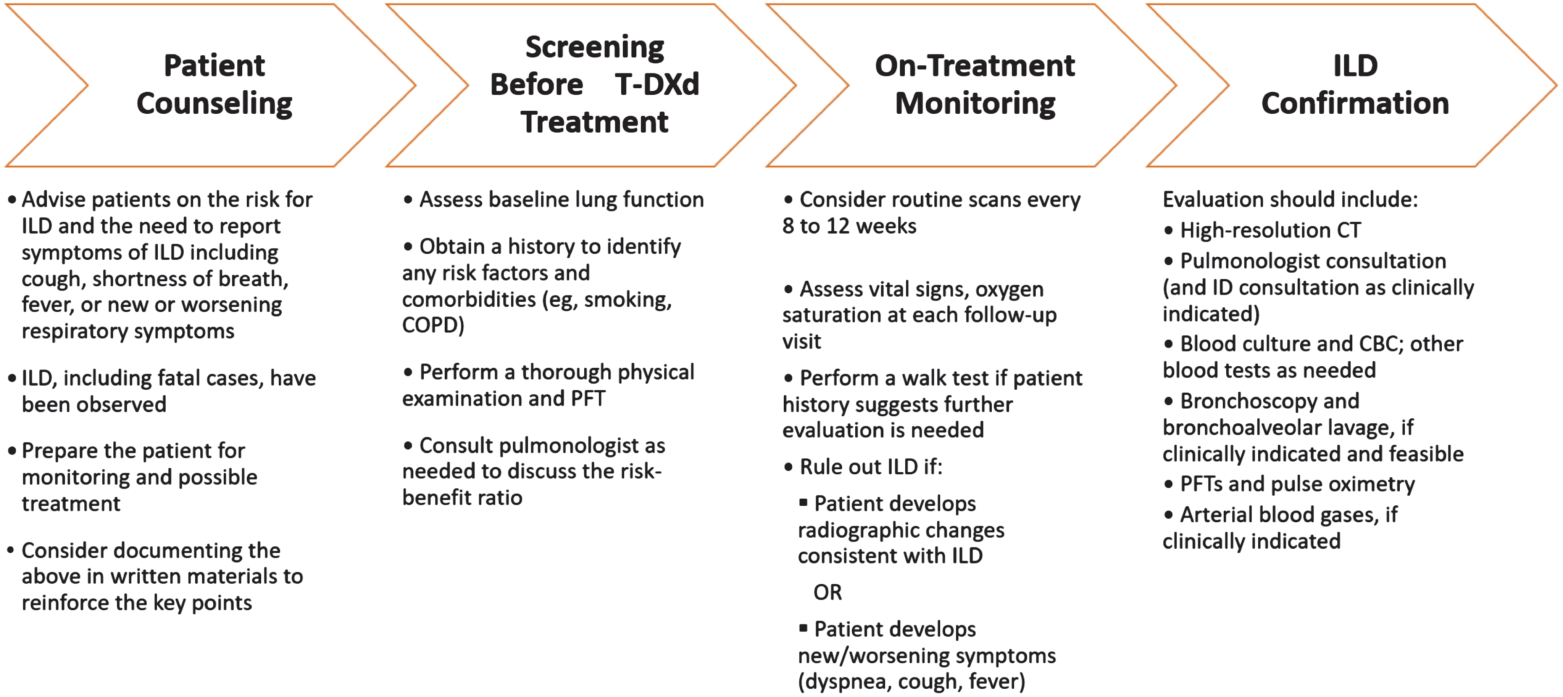
ILD associated with T-DXd treatment: patient counseling, pretreatment screening, on-treatment monitoring, and confirmation. Abbreviations: CBC, complete blood count; COPD, chronic obstructive pulmonary disease; CT, computed tomography; ID, infectious disease; ILD, interstitial lung disease; PFT, pulmonary function test; PK, pharmacokinetic; T-DXd, trastuzumab deruxtecan.

In the August 2019 data analysis of the DESTINY-Breast01 study, ILD of any grade was observed in 13.6% (*n* = 25) of patients. These events were primarily National Cancer Institute Common Terminology Criteria for Adverse Events grade 1 or 2 (10.9%) but led to death in 4 patients (2.2%). Patients with a history of (noninfectious) ILD/pneumonitis that required steroids, current ILD/pneumonitis, or suspected ILD/pneumonitis that cannot be ruled out by imaging at screening were excluded from the study.^9^ Of the 4 fatal cases, 2 patients were smokers or former smokers and 3 had baseline lung metastases. The grade of onset of ILD varied and time to onset was from 63 to 148 days. Three patients received steroids as a part of treatment.^22^ Further analyses on risk factors for ILD are still underway. In the updated data analysis that accounted for 15 additional months of follow-up (March 2021), 4 new ILD cases were reported (15.8%, Table 1) and were classified as grade 1 (3.8%), 2 (8.7%), and 5 (2.7%).^11^ Guidelines and consensus statements on drug-induced ILD are available from professional congresses and societies,^23,24^ but these guidelines do not discuss HER2-targeted therapies. Regardless, proactive ILD monitoring strategies are invariably warranted, routinely practiced, and strongly encouraged. Fig. 2 outlines an approach to counseling, patient selection, monitoring, and diagnosis of ILD based on authors’ clinical practices and the T-DXd clinical trial program.

The risk of ILD as a potentially irreversible and fatal AE is an important point to address during discussions with patients about treatment selection. Baseline pulmonary assessments, vigilant monitoring, and prompt management may reduce the risk for the development of severe ILD. Patients should have a clinical history and physical examination completed to clarify any underlying respiratory issues before embarking on T-DXd therapy. This evaluation may identify concerns regarding the patient’s pulmonary function tests (PFTs), history of chronic obstructive pulmonary disease, pre-existing ILD, and smoking history. Pulse oximetry can be used to noninvasively measure the patient’s oxygen saturation, which should ideally be 90%-95% or higher on room air. Although shortness of breath may not be present, hypoxia warrants further evaluation. Patients with compromised baseline pulmonary function are likely to be treated by a pulmonologist, who should be consulted. Although compromised lung function does not necessarily preclude treatment with T-DXd, it should be considered when assessing the benefit-to-risk ratio of treatment.

Once T-DXd treatment is initiated, frequent monitoring for ILD is recommended. Seemingly minor symptoms can be the first indication of ILD; thus, patients should be instructed to immediately report any indicative signs and symptoms, including cough, fever, shortness of breath, or any new or worsening respiratory symptoms. In the metastatic setting, routine restaging scans (computed tomography [CT] scan of thorax, abdomen, and pelvis or PET-CT) are performed every 8-12 weeks as part of disease monitoring at our institutions and also serve as an opportunity to check for signs of lung injury (eg, honeycombing, traction bronchiectasis) even if a patient is asymptomatic. In clinical trials, CT imaging was performed every 6 weeks which may have helped to detect asymptomatic ILD.^9^ Some clinicians find it useful to schedule PFTs, to assess decreased DLCO (diffusing capacity of the lungs for carbon monoxide), in between scans (ie, every 3 months, alternating with CT/PET scans) and have incorporated this into their institutional protocols. Vital signs, including pulse oximetry, are also assessed at each clinic visit prior to administration of T-DXd and may be indicative of pulmonary function. If needed, a walk test in the hallway, which involves walking the patient while measuring their oxygen saturation to detect decreases, can be performed to assess worsening respiratory symptoms, document desaturation, and evaluate the need for supplemental oxygen. However, the authors acknowledge that the use of measures such as PFTs, exercise oximetry, or DLCO for the detection of grade 1 ILD have not been studied. Routine clinic visits with the medical oncologist are another opportunity to ask patients about new or worsening respiratory symptoms that they may have been hesitant to proactively address.

If the patient is experiencing pulmonary symptoms, and ILD is suspected, further workup should be performed to rule out the causes of the symptoms (Fig. 2), which could be related to COVID-19 or other infection, pulmonary embolism, or progression of lung metastases. Management may differ depending on the origin of the condition. If ILD associated with T-DXd treatment is confirmed, T-DXd should be held or discontinued, depending on the grade of ILD. In addition, prompt administration of corticosteroids and supportive care are recommended in accordance with the T-DXd Prescribing Information^7^ (Table 2). Early data have shown that prompt identification and aggressive management of ILD, as recommended in Table 2, may reduce the risk of high-grade ILD.

## Risk of Cardiotoxicity (Decreased LVEF)

As previously discussed, T-DXd was not associated with clinically significant cardiomyopathy in the DESTINY-Breast01 study. In the updated June 2020 data analysis, there were 4 total events of LVEF decrease (3 grade 2 and 1 grade 3).^17^ Regardless, decreased LVEF is a known risk associated with HER2-targeted therapies. As such, baseline left ventricular function should be assessed prior to T-DXd administration with an echocardiogram with strain imaging. Repeat echocardiograms with strain imaging should then be obtained during regular intervals while T-DXd treatment continues (Table 2). Monitoring intervals are typically based on provider preference and patient-specific factors. Initial follow-up echocardiograms are commonly repeated every 3 months; some clinicians may extend repeat testing to 6-month intervals if the patient is stable, is asymptomatic, and has demonstrated no history of or recent LVEF decreases.

If decreased LVEF is detected, the T-DXd Prescribing Information recommends dosage modifications/interruptions and therapy discontinuation depending on the degree of decrease (Table 2). The involvement of the cardiologist can provide insight into risk stratification and initiation and monitoring of treatments needed to reduce cardiac afterload. Cardio-Oncology or cardiologists who specialize in the detection, monitoring, and treatment of cardiovascular disease occurring as an AE of cancer treatment,^25^ has become an emerging field because cardiac AEs are common among different types of anticancer therapies. These teams, however, are not available at all institutions. Regardless, the goal is to manage these patients in a multidisciplinary team setting with experts in cardiac function to enable the proper anti-cancer treatment to continue, if possible.

## Other AEs

Additional AEs may also be observed with T-DXd therapy. For example, the risk of alopecia is common with anticancer treatments, including T-DXd. It is best to be forthcoming with patients about this potential AE. Treatment experience with T-DXd has indicated that alopecia more frequently manifests as hair thinning, rather than hair loss. Cold cap therapy may be used in an attempt to prevent alopecia; however, the efficacy of this therapy is unknown with T-DXd, and studies are ongoing. Furthermore, it is not always appropriate or practical to use in the metastatic setting, such as when there are known metastases to the skull.

Patients receiving treatment with T-DXd may also experience fatigue. However, it can be difficult to differentiate disease-related fatigue from fatigue associated with T-DXd treatment. Regardless of the cause, fatigue can interfere with patient activities of daily living but is usually manageable. Patients may be receptive to trying light exercise, such as yoga, and diet modification may help ensure patients receive adequate nutrients. Pharmacological interventions (eg, methylphenidate) can be considered but remain investigational^26^ and have not been studied in combination with T-DXd. Clinical judgment is advised.

## Summary and Conclusions

**Figure 3.**
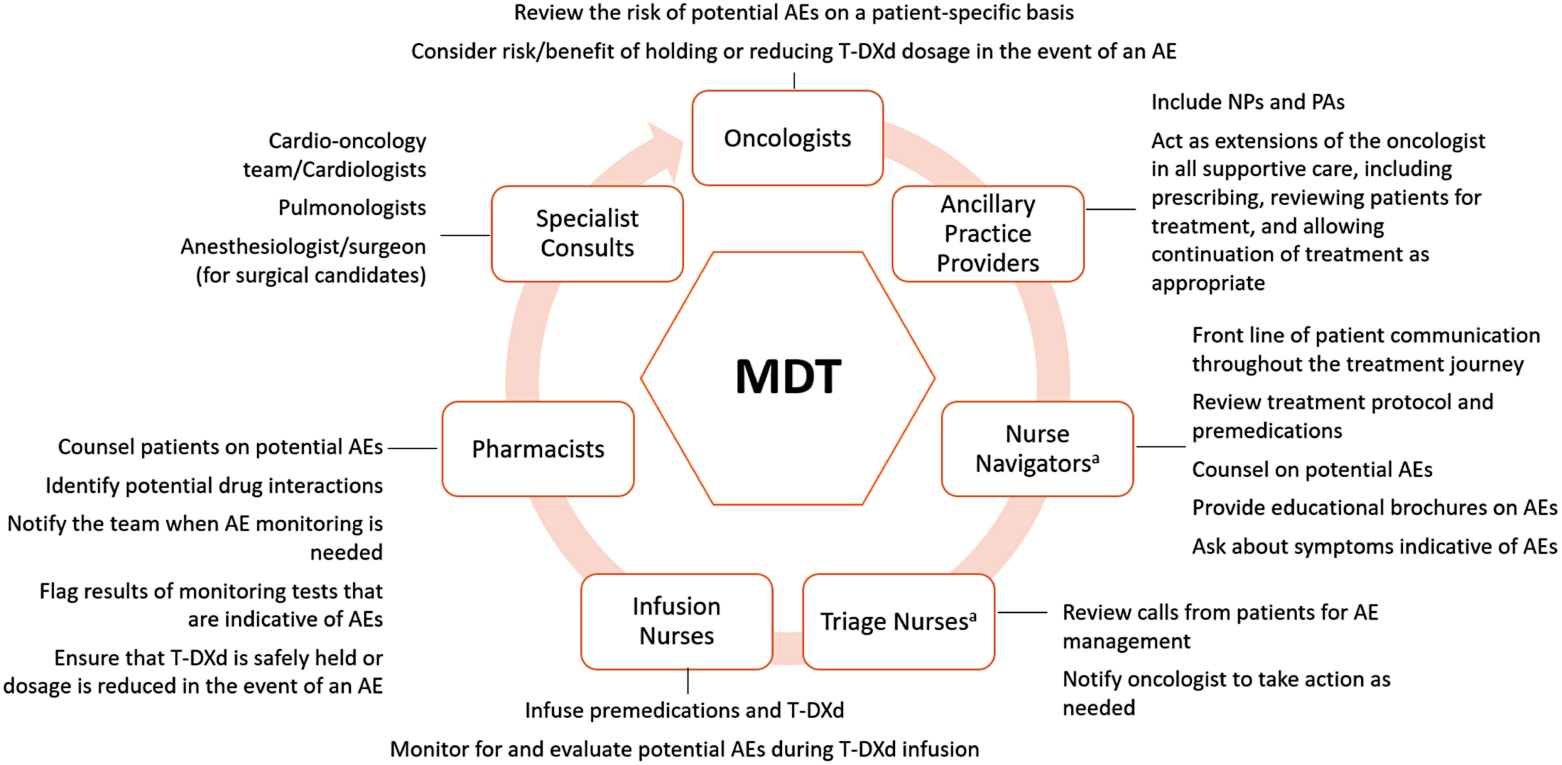
The multidisciplinary approach to preventing, monitoring for, and managing AEs associated with T-DXd therapy. ^a^The roles of the nurse navigators and triage nurses described here may interchange or overlap, depending on the institution. Abbreviations: AE, adverse event; MDT, multidisciplinary team; NP, nurse practitioner; PA, physician assistant; T-DXd, trastuzumab deruxtecan.

T-DXd is highly efficacious in clinical studies. When administered to a heavily pretreated population of patients with HER2-positive mBC, T-DXd demonstrated a confirmed ORR of 62.0%, median DOR of 18.2 months, and median PFS of 19.4 months (DESTINY-Breast01, March 2021 data analysis).^11^ The AEs associated with T-DXd treatment are not new to patients who are candidates for this drug. Despite the familiarity of these AEs, patient counseling to ensure a thorough understanding of the risks and benefits is essential to maximize the benefit of therapy. In our clinical experience, N/V has the biggest impact on patient quality of life. We have most frequently observed N/V and neutropenia in our patients treated with T-DXd, which aligns with clinical study data. Our oncology teams have become skilled at preventing and monitoring these AEs and consider them manageable. A multidisciplinary approach to AE management assures that expert perspectives are delivered from all angles (Fig. 3). With proactive measures, proper vigilance, and effective treatment, AEs associated with T-DXd treatment are generally manageable, allowing patients the opportunity to receive the benefit of T-DXd treatment for HER2-positive mBC.

## Data Availability

No new data were created or analyzed in the development of this review article.
